# MicroRNA-34a regulates doxorubicin-induced cardiotoxicity in rat

**DOI:** 10.18632/oncotarget.11468

**Published:** 2016-08-22

**Authors:** Elena Piegari, Rosa Russo, Donato Cappetta, Grazia Esposito, Konrad Urbanek, Carmela Dell'Aversana, Lucia Altucci, Liberato Berrino, Francesco Rossi, Antonella De Angelis

**Affiliations:** ^1^ Department of Experimental Medicine, Section of Pharmacology, Second University of Naples, Naples, Italy; ^2^ Institute of Genetics and Biophysics, IGB ‘Adriano Buzzati-Traverso’, Naples, Italy; ^3^ Department of Biochemistry, Biophysics and General Pathology, Second University of Naples, Naples, Italy

**Keywords:** miR-34a, doxorubicin-induced cardiotoxicity, rat cardiac progenitor cells, SIRT1, cellular senescence

## Abstract

New strategies to prevent and early detect the cardiotoxic effects of the anticancer drug doxorubicin (DOXO) are required. MicroRNAs emerged as potential diagnostic, therapeutic and prognostic approaches in cardiovascular diseases. MiR-34a has a role in cardiac dysfunction and ageing and is involved in several cellular processes associated with DOXO cardiotoxicity. Our *in vitro* and *in vivo* results indicated that after DOXO exposure the levels of miR-34a are enhanced in cardiac cells, including Cardiac Progenitor Cells (CPCs). Since one of the determining event responsible for the initiation and evolution of the DOXO toxicity arises at the level of the CPC compartment, we evaluated if miR-34a pharmacological inhibition in these cells ameliorates the detrimental aftermath of the drug. AntimiR-34a has beneficial consequences on vitality, proliferation, apoptosis and senescence of DOXO-treated rat CPC. These effects are mediated by an increase of prosurvival miR-34a targets Bcl-2 and SIRT1, accompanied by a decrease of acetylated-p53 and p16^INK4a^. Importantly, miR-34a silencing also reduces the release of this miRNA from DOXO-exposed rCPCs, decreasing its negative paracrine effects on other rat cardiac cells. In conclusion, the silencing of miR-34a could represent a future therapeutic option for cardioprotection in DOXO toxicity and at the same time, it could be considered as a circulating biomarker for anthracycline-induced cardiac damage.

## INTRODUCTION

Anthracyclines, a class of chemotherapeutic drugs widely used to treat several pediatric and adult tumors, are among the leading causes of cardiotoxicity in cancer survivors. The spectrum of short- and long-term cardiotoxic effects induced by doxorubicin (DOXO), one of the most effective anthracycline, ranges from subclinical ventricular dysfunction to severe cardiomyopathy and heart failure that may result in cardiac transplantation or death [1, 2]. Therefore, there is an urgent need to early diagnose DOXO-induced toxicity and prevent future ventricular complications.

Recently, microRNAs (miRNAs) have been recognized as fundamental regulators of cardiovascular functions and diseases and currently there is a growing interest in develop their potential as both biomarkers and therapeutics for cardiovascular pathologies [3–7]. MiRNAs are small (~22 nucleotides), noncoding RNAs that regulate gene expression, primarily inhibiting the translation of a specific mRNA target into a functional protein and/or promoting mRNA degradation [8]. They can also convey part of the biological activity of conditioned media from cell culture and their presence in body fluids supports their role in cell-to-cell information transfer [5, 7].

MiR-34 family members (miR-34a, -34b, and -34c) are upregulated in the heart in response to stress (i.e. myocardial infarction) and contribute to the age-dependent decline in cardiac function [9, 10]. In particular, miR-34a the one predominantly expressed in the heart [10] modulates several target proteins involved in cell cycle, apoptosis, senescence, differentiation and cellular development [11]. Pharmacological inhibition of miR-34a improves cardiac regeneration and function in experimental models of myocardial infarction and pressure overload-induced hypertrophy [9, 10, 12], suggesting the silencing of this miRNA as a future therapeutic option for cardioprotection. Therefore, we evaluated whether miR-34a contributes to the onset of DOXO-induced cardiotoxicity. In particular, based on our previous studies that have demonstrated that detrimental effects of DOXO on cardiac progenitor cells (CPCs), negatively affect myocardial homeostasis [13, 14, 15], we investigated the possibility that miR-34a expression is modified in rat CPCs (rCPCs) after DOXO exposure. Additionally we address the possibility that modulation of this miRNA could protect rCPCs from anthracycline toxicity.

Moreover, a recent study revealed an increased expression of miR-34a in mouse heart as an early molecular event of cardiac tissue injury during DOXO treatment [16]. Interestingly, miR-34a has been identified as a predictive plasma marker of future heart failure in patients after acute myocardial infarction [17]. These findings prompted us to determine whether miR-34a could be a potential circulating biomarker of DOXO-induced cardiac damage.

## RESULTS

### MiR-34a expression and release in DOXO-treated rat cardiac cells

**Figure 1:**
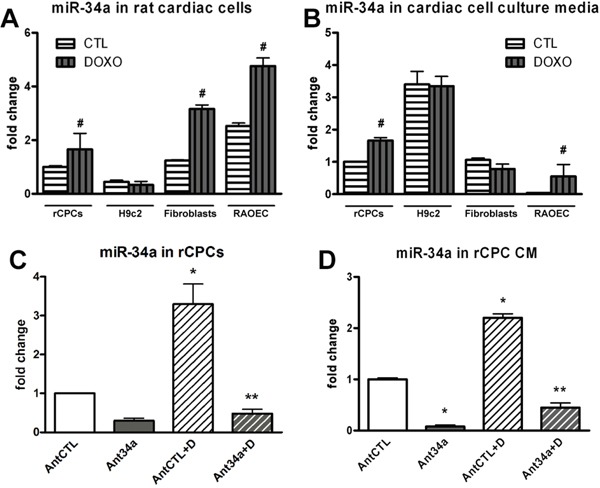
MiR-34a expression in DOXO-exposed cardiac cells **A.** qPCR analysis of intracellular miR-34a levels in rCPCs, H9c2, fibroblasts and RAOECs after treatment with 0.5 μM DOXO for 24h. **B.** The release of miR-34a was evaluated by qPCR in cardiac cell culture media after DOXO exposure. **C.** MiR-34a was silenced by Ant34a and its levels were measured by qPCR after DOXO treatment. **D.** MiR-34a levels, quantified by qPCR in rCPC conditioned media (CM). Results are presented as mean ± SD. MiRNA expression is shown as fold change with respect to rCPC CTL (panels A and B) or AntCTL (panels C and D). ^#^p<0.05 vs CTL; *p<0.05 vs AntCTL; **p<0.05 vs AntCTL+D. Ant34a: antimiR-34a; AntCTL: Ant34a negative control; D: DOXO.

To determine whether miR-34a take part to the complex mechanisms involved in DOXO-induced cardiotoxicity, its expression was evaluated in rat cardiac cells. Rat CPCs, H9c2 cells, fibroblasts and RAOEC (rat aortic endothelial cells) were exposed to 0.5 μM DOXO for 24h. Real time PCR (qPCR) results showed a significant upregulation of miR-34a in all cardiac cells after DOXO treatment except for H9c2 (Figure 1A). Nevertheless, miR-34a expression was significantly increased in these cells after 48h of DOXO exposure (Supplementary Figure 1). Moreover, to investigate whether miR-34a could be released by cardiac cells, the secretion of this miRNA in cell culture media was evaluated. MiR-34a levels significantly increased only in media from DOXO-treated rCPC and RAOEC but not in H9c2 and fibroblast media (Figure 1B).

### MiR-34a inhibition in DOXO-exposed rCPCs

The findings that the damage at the level of CPC population is responsible of DOXO-induced cardiotoxicity [13, 14, 15], prompted us to consider whether miR-34a inhibition in rCPCs could provide therapeutic benefit to DOXO toxicity. The cells were transfected with antimiR-34a (Ant34a) 24h before DOXO administration and miR-34a levels were measured by qPCR. Ant34a negative control (AntCTL), a random sequence antimiR molecule that has been validated to produce no identifiable effects on known miRNA function, was used as control. MiR-34a, both produced and secreted by rCPCs, was drastically silenced by Ant34a, as well in the absence and presence of DOXO (Figure 1C and 1D).

### AntimiR-34a effects on vitality and proliferation of DOXO-treated rCPCs

**Figure 2:**
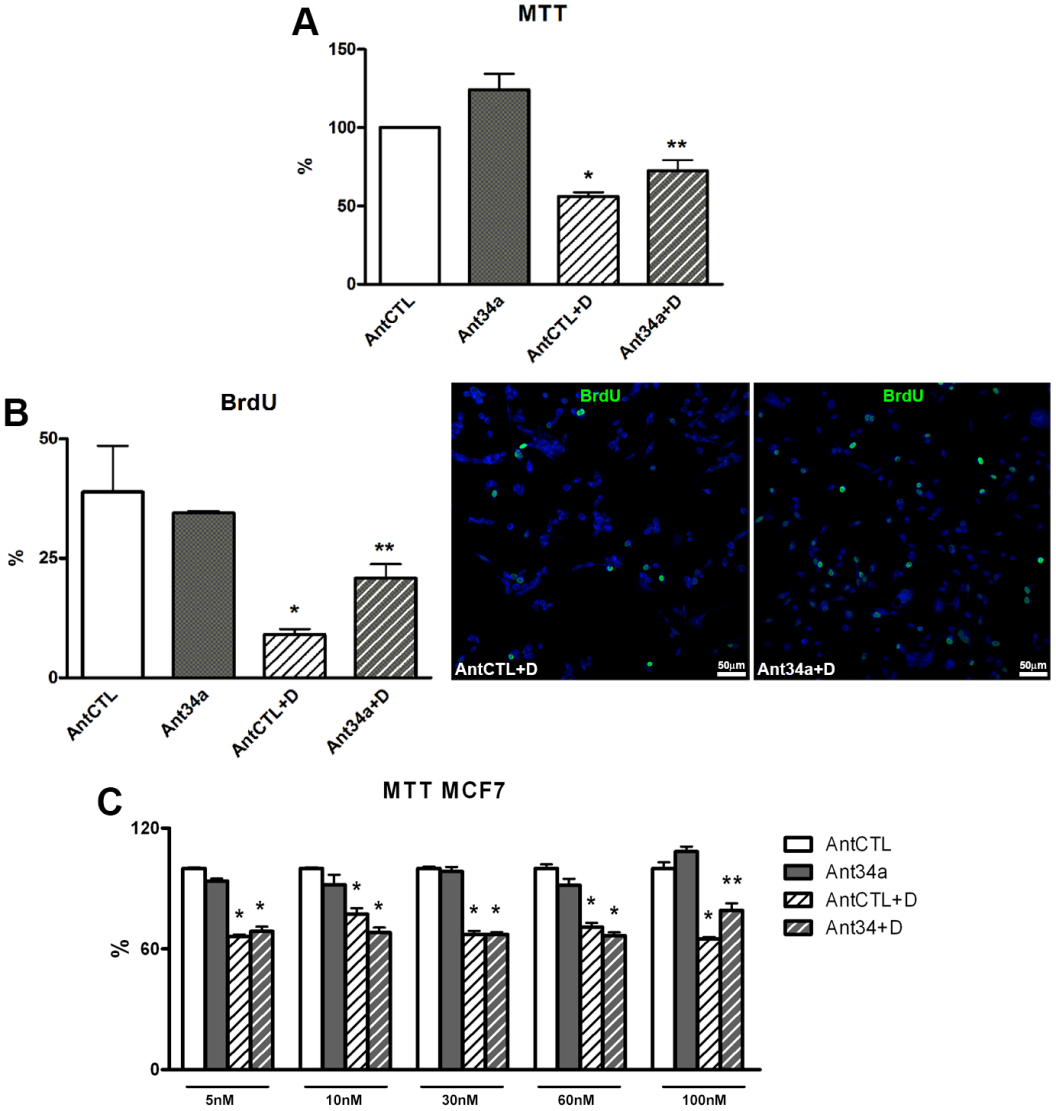
Effect of Ant34a on DOXO-treated rCPCs and cancer cells **A.** rCPC viability was determined by MTT assay after miR-34a inhibition followed by DOXO exposure. **B.** BrdU incorporation assay shows the rate of rCPCs proliferation after treatment with Ant34a and DOXO. BrdU positive cells (green) were showed in representative pictures, nuclei were counterstained with DAPI (blue). **C.** Effects of Ant34a increasing concentrations on vitality of MCF7 exposed to 0.5 μM DOXO for 24h. Results are presented as mean ± SD. *p<0.05 vs AntCTL; **p<0.05 vs AntCTL+D. Ant34a: antimiR-34a; AntCTL: Ant34a negative control; D: DOXO.

As expected the vitality and the proliferation of AntCTL+DOXO rCPCs markedly decreased respect to AntCTL treated cells (Figure 2A and 2B). Importantly, miR-34a inhibition was able to reduce the cytotoxic effect of DOXO. In fact, Ant34a treatment significantly increased rCPC vitality and proliferation after DOXO exposure as measured by MTT assay and the rate of BrdU incorporation respectively (Figure 2A and 2B). In particular, in presence of DOXO, miR-34a inhibition increased vitality by 29% and the percentage of BrdU positive rCPCs raised more than 2-fold with Ant34a+DOXO treatment with respect to AntCTL+DOXO (Figure 2A and 2B).

### AntimiR-34a effects on DOXO-treated cancer cells

To determine whether miR-34a inhibition could interfere with anthracycline antitumor activity, the effects of increasing concentration of Ant34a were evaluated on breast cancer cell line (MCF7) exposed to DOXO. In this settings, 10 nM Ant34a treatment, that increased vitality of DOXO-treated rCPCs (Figure 2A), did not influence cytotoxic action of drug on tumor cells (Figure 2C). However, the reduction of the cytotoxic effect of DOXO in MCF7, was observed with a 10-fold higher Ant34a concentration (Figure 2C).

### Role of miR-34a in apoptosis of DOXO-exposed rCPCs

**Figure 3:**
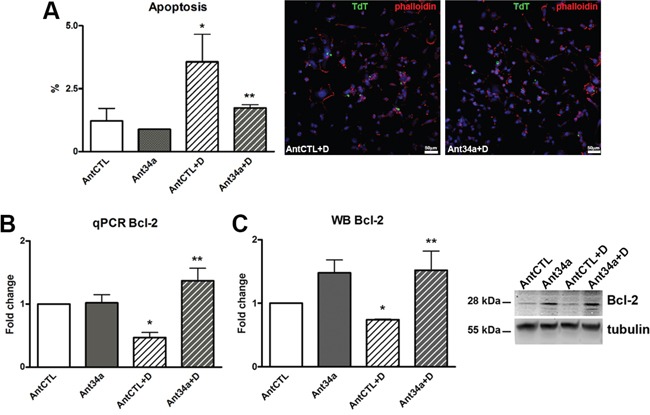
Role of miR-34a in apoptosis of DOXO-exposed rCPCs **A.** Apoptosis was evaluated by TdT assay in rCPCs treated with Ant34a and DOXO; apoptotic cells (green) were showed in representative pictures, nuclei were counterstained with DAPI (blue) and cell cytoskeleton with fluorochrome-conjugated phalloidin (red). **B.** Bcl-2 mRNA levels were analysed by qPCR. **C.** Western blot analysis shows Bcl-2 protein levels. Results are presented as mean ± SD. mRNA and protein expressions are shown as fold change with respect to AntCTL. *p<0.05 vs AntCTL; **p<0.05 vs AntCTL+D. Ant34a: antimiR-34a; AntCTL: Ant34a negative control; D: DOXO.

As miR-34a has been shown to prompt apoptosis in tumor cells [18] and recently also in cardiomyocytes [10, 12], and apoptosis is an essential process in DOXO cardiotoxicity [19, 20], we next evaluated whether miR-34a has a role in DOXO-induced apoptosis in rCPCs. TdT assay showed that the number of apoptic cells were markedly reduced by inhibition of miR-34a in DOXO-treated rCPCs (51% vs AntCTL+DOXO) (Figure 3A). To assess if the protection from cell death is mediated by Bcl-2, a target of miR-34a, the expression of this antiapoptic protein was measured. Ant34a administration significantly increased Bcl-2 mRNA and protein levels in DOXO rCPCs as validated by qPCR and western blot analysis respectively (Figure 3B and 3C). In particular, Bcl-2 mRNA and protein levels in Ant34a+DOXO cells were respectively almost 3-fold and 2-fold higher with respect to AntCTL+DOXO.

### p53-miR-34a-SIRT1 axis in DOXO-exposed rCPCs

**Figure 4:**
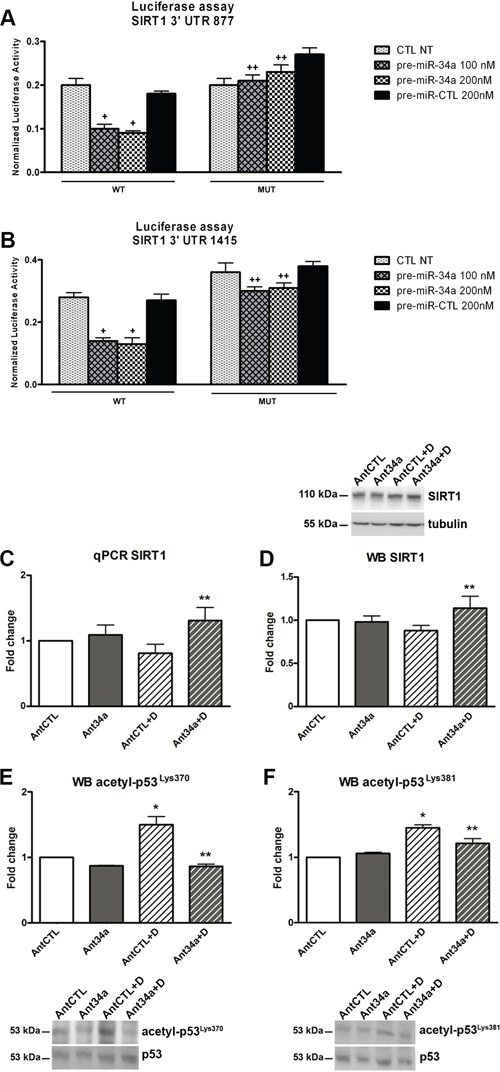
MiR-34a modulation of SIRT1-p53 pathway in DOXO-exposed rCPCs **A.** Luciferase activity assay for SIRT1 3′UTR 877 and **B.** SIRT1 3′UTR 1415 at 24h. Results are expressed as normalized luciferase activity. **C.** SIRT1 mRNA levels analysed by qPCR. **D.** Western blot analysis shows SIRT1 protein levels. **E.** Acetyl-p53^Lys370^ expression levels were evaluated by western blot. **F.** Analysis of acetyl-p53^Lys381^ expression levels. Results are presented as mean ± SD. mRNA and protein expressions are shown as fold change with respect to AntCTL. ^+^p<0.05 vs CTL NT; ^++^p<0.05 vs corresponding WT conditions; *p<0.05 vs AntCTL; **p<0.05 vs AntCTL+D. CTL NT: no treated control; WT: Wild Type; MUT: mutated; Ant34a: antimiR-34a; AntCTL: Ant34a negative control; D: DOXO.

SIRT1, one of several miR-34a targets, is a member of the Sirtuin family of class III histone deacetylases, which regulates cell cycle, cellular survival, senescence and metabolism [21, 22]. It has been reported that SIRT1 plays an important regulatory role in the connection between DNA damage, p53 and miR-34a [11]. P53 is linked to miR-34a in a multifaceted manner and the most common identified pathway is SIRT1, which deacetylates its non-histonic substrate p53. Practically, a positive feedback loop regulates p53-miR-34a-SIRT1 axis: p53 induces miR-34a and miR-34a activates p53 by inhibiting the expression SIRT1 [11, 23]. As previously demonstrated, DOXO induces acetylation of p53 in human CPCs promoting cell cycle arrest, apoptosis or senescence [24]. Therefore, we investigate whether miR-34a inhibition could modulate SIRT1 and p53 expression and function in DOXO-treated rCPCs. The 3′ untranslated region (UTR) luciferase assay confirmed that SIRT1 is a direct target of miR-34a. In fact, transfection of miR-34a precursors clearly reduced luciferase activity for the wild-type reporter but did not for the mutant types SIRT1 3′UTR both at 24h (Figure 4A and 4B) and 48h (Supplementary Figures 2A and 2B). Consistently, Ant34a treatment upregulated the expression of both SIRT1 mRNA (62% vs AntCTL+DOXO) and protein (30% vs AntCTL+DOXO) after DOXO exposure of rCPCs (Figure 4C and 4D). Moreover, in our experimental settings, miR-34a inhibition decreased DOXO-induced p53 acetylation. Both acetyl-p53^Lys370^ and acetyl-p53^Lys381^ were significantly reduced subsequently to Ant34a treatment (Figure 4E and 4F), consistent with SIRT1 upregulation.

### AntimiR-34a effects on senescence of DOXO-treated rCPCs

**Figure 5:**
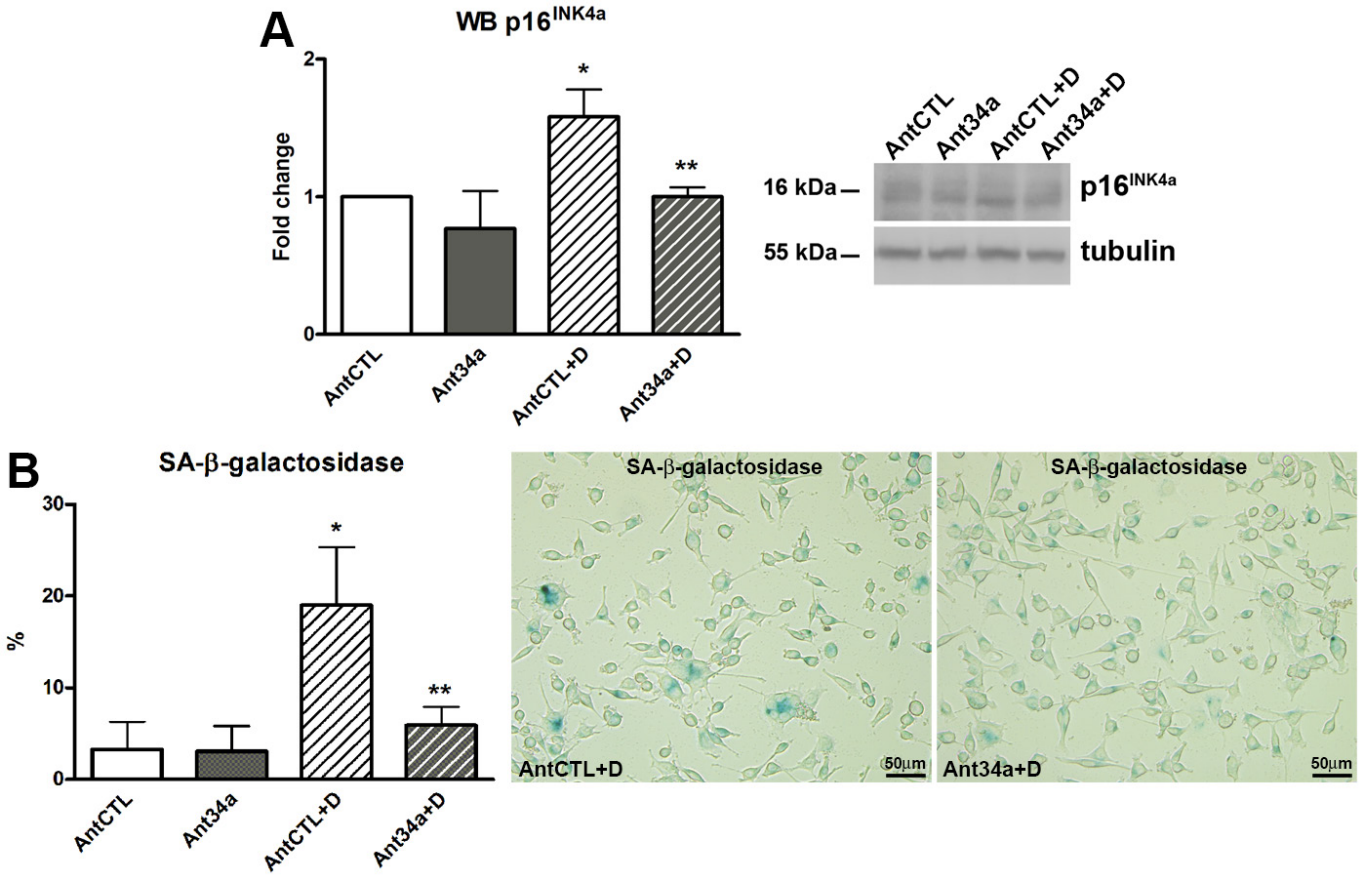
MiR-34a involvement in cell senescence of DOXO-treated rCPCs **A.** Western blot analysis of p16^INK4a^. **B.** SA-β-galactosidase assay was performed to identify senescent rCPCs after miR-34a inhibition and DOXO exposure; senescent cells (light blue) were showed in representative pictures. Results are presented as mean ± SD. Protein expressions are shown as fold change with respect to AntCTL. *p<0.05 vs AntCTL; **p<0.05 vs AntCTL+D. Ant34a: antimiR-34a; AntCTL: Ant34a negative control; D: DOXO.

The inactivation of p53, by SIRT1-mediated deacetylation, may influence the process of cellular senescence [25]. In rCPCs, DOXO induced an up-regulation of p16^INK4a^ and led to a massive cellular senescence, confirming previously published results [13, 14, 24]. However, with respect to AntCTL+DOXO, Ant34a+DOXO treatment resulted in a significant reduction in p16^INK4a^ expression (37%) (Figure 5A). These data were confirmed by a substantial decrease of senescent cells (69% reduction vs AntCTL+DOXO), as demonstrated by the reduced percentage of SA-β-galactosidase positive rCPCs (Figure 5B). Thus, miR-34a inhibition could protect rCPCs from DOXO-induced senescence.

### Paracrine role of miR-34a on rat cardiac cells

**Figure 6:**
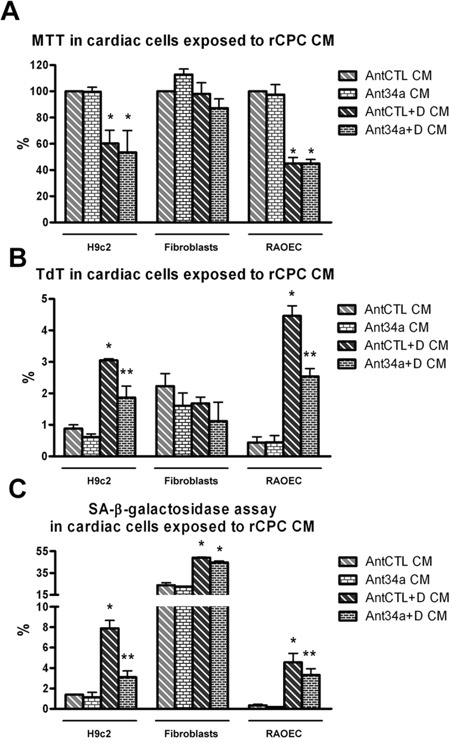
Effects of rCPC conditioned media (CM) on cardiac cells **A**. Cell viability was evaluated by MTT in rat cardiac cells exposed for 48h to rCPC CM. **B**. TdT assay showed the percentage of apoptosis in CM-treated cardiac cells. **C.** SA-β-galactosidase assay in cardiac cells exposed to rCPC CM. *p<0.05 vs AntCTL CM; **p<0.05 vs AntCTL+D CM.

Since in rCPCs DOXO stimulates also miR-34a secretion, we investigated its possible paracrine effects on other cardiac cell types. For this purpose, H9c2, fibroblasts and RAOEC were grown for 48h in conditioned media (CM) from AntCTL+DOXO- or Ant34a+DOXO-treated rCPCs. In H9c2 and RAOEC, although cell vitality was significantly reduced in all samples exposed to DOXO CM, no significant variation was observed between AntCTL+DOXO CM and Ant34a+DOXO CM treated cells (Figure 6A). Nevertheless, with respect to AntCTL+DOXO CM, the exposure to Ant34a+DOXO CM caused a significant reduction of both apoptotic and senescent H9c2 and endothelial cells, as demonstrated by TdT and SA-β-galactosidase assays respectively (Figure 6B and 6C). On the contrary, vitality, apoptosis and senescence of cardiac fibroblasts were not influenced by Ant34a+DOXO CM treatment (Figures 6A, 6B and 6C). These results indicated that miR-34a released by DOXO-treated rCPCs can have negative consequences also on other cardiac cells in a paracrine manner. Notably pharmacological inhibition of miR-34a could revert these effects.

### Evaluation of miR-34a levels in tissues and plasma of rats affected by DOXO-induced cardiomyopathy

**Figure 7:**
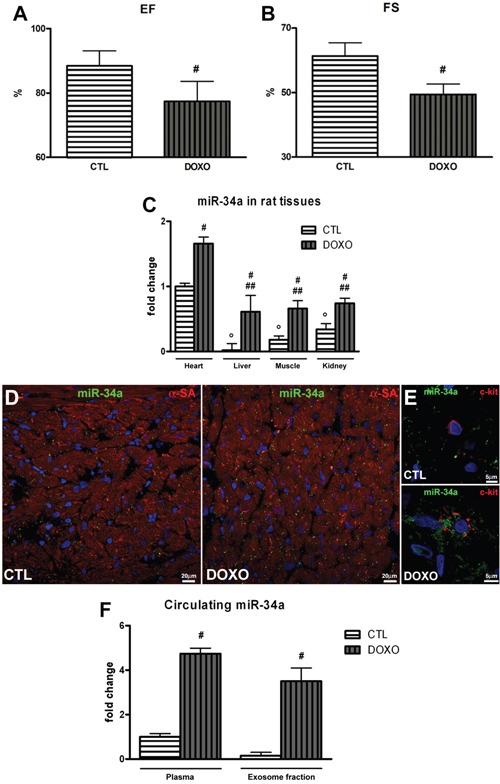
Cardiac function and miR-34a levels in DOXO-treated rats **A.** Ejection fraction and **B.** Fractional shortening were evaluated by echocardiographic measures in DOXO-cardiomyopathic rats. Rats received 6 intraperitoneal injections of 2.5 mg/kg of DOXO over a period of 2 weeks (cumulative dose 15 mg/kg) and cardiac function was evaluated at 3 weeks after the first injection of DOXO. **C.** qPCR analysis indicated miR-34a expression in tissues of DOXO-treated rats sacrificed at 3 weeks. **D.** In situ hybridization with digoxigenin-labeled miR-34a probe in heart sections of DOXO-treated rats. MiR-34a is visualized in green, cardiomyocytes in red (α-SA: α-sarcomeric actin) and nuclei in blue (DAPI). **E.** Representative image of a higher magnification picture of c-kit-positive rCPC.MiR-34a is visualized in green, c-kit in red and nuclei in blue (DAPI). **F.** Plasma and exosome levels of miR-34a were evaluated by qPCR. Results are presented as mean ± SD. MiRNA expression is shown as fold change with respect to heart CTL (panel C) or plasma CTL (Panel H). °p<0.05 vs heart CTL; ^#^p<0.05 vs CTL (panels A, B and F); ^#^p<0.05 vs tissue CTL (panel C); ^##^p<0.05 vs heart DOXO.

Following the demonstration that *in vitro* miR-34a secretion by rCPCs and endothelial cells is enhanced after DOXO exposure, we evaluated if, also *in vivo*, DOXO triggers the expression of miR-34a in heart tissue. MiR-34a levels in liver, kidney and skeletal muscle were also analysed. The well-established animal model of DOXO-induced cardiomyopathy was represented by F344 rats treated with a cumulative dose of 15 mg/kg DOXO [13, 24, 26]. At 3 weeks after the first injection of DOXO, cardiac functions were measured by echocardiography and then organs and blood samples were collected. DOXO-treated animals showed left ventricular dysfunction respect to controls, in particular ejection fraction (EF) and fractional shortening (FS) decreased by 10% and 20% respectively (Figure 7A and 7B). qPCR results indicated that DOXO administration upregulated miR-34a in heart, liver, kidney, skeletal muscle (Figure 7C). However, in DOXO-treated animals the heart contribution seems to be predominant since, with respect to the other organs, miR-34a levels were higher in cardiac tissue (Figure 7C). Moreover, corroborating *in vitro* experiments, miR-34a *in situ* hybridization in heart sections of DOXO-treated animals showed higher levels of this miRNA in cardiac cells, including c-kit positive rCPCs (Figure 7D and 7E). Importantly, plasma and exosome fraction from rats affected by DOXO-induced cardiomyopathy were highly enriched in miR-34a respect to control animals. MiR-34a levels increased 4.7-fold in plasma and 3.5-fold in exosome fraction, indicating that it is mainly released into the blood within exosomes (Figure 7F). Therefore, cardiac cells could release miR-34a in the peripheral circulation and it could be potentially used as a marker of DOXO-induced cardiac damage.

## DISCUSSION

The present study had provided two major findings. First, miR-34a increases in rat cardiac cells exposed to DOXO and its inhibition in rCPCs can partially prevent the negative effects driven by DOXO not only in these cells but also in neighboring ones. Second, in the light of its increasing levels in heart and plasma of DOXO-cardiomyopathic rats, miR-34a could be a potential circulating biomarker of anthracycline induced cardiac damage.

Cardiotoxicity remains the major side effect of anthracycline, therefore the search for a strategy able to counteract chemotherapy-induced cardiac complications is extremely important, above all in view of the growing population of cancer survivors. The involvement of miRNAs, a class of small non-coding regulatory RNAs, in almost all processes underlying cardiovascular disease raises the exciting possibility for the therapeutic applications of these molecules [3, 6, 27].

In this paper, we have focused our attention on miR-34a, a miRNA involved in several cellular processes implicated in DOXO cardiotoxicity, such as apoptosis and senescence [11] and recently recognized as a key regulator in cardiac dysfunction and ageing [9, 10, 12, 17]. Our data indicate that DOXO exposure upregulates, both *in vitro* and *in vivo*, miR-34a expression in rat cardiac cells and increases its release from rCPCs and endothelial cells, supporting the hypothesis that this miRNA could be the possible mediator of the negative consequences driven by DOXO. Since previous studies have demonstrated that resident CPCs are very sensitive to DOXO that severely impairs their functions and regenerative capacity *in vitro* and *in vivo* [13, 14, 24], influencing the subsequent and progressive cardiac damage, we verified if miR-34a pharmacological silencing in these cells prevents anthracycline toxicity. In DOXO-treated rCPCs miR-34a inhibition increased vitality and proliferation and reduced apoptosis and senescence. Notably, miR-34a released by DOXO-treated rCPCs has negative paracrine consequences on other cardiac cells and its inhibition could revert these effects. Different cardiac cells including CPCs, cardiomyocytes and endothelial cells, have been demonstrated to secrete miRNAs, suggesting a role of these small RNAs to act as paracrine signalling mediators in several cardiovascular diseases [17, 28–30]. In this view, the silencing of a dysregulated miRNA may represent a novel strategy, not only for modulating the intracellular expression of the target miRNA but also for reducing secreted miRNA, abrogating pathological cell-to-cell communication.

To further assess the mechanisms which underlie the above miR-34a mediated processes in DOXO toxicity, we examined the expression of some direct or indirect miR-34a targets, which have previously been linked to cellular survival, apoptosis and senescence. Among miR-34a direct targets, Bcl-2 and SIRT1 expression was evaluated. Bcl-2, an antiapoptotic protein that promotes cell survival in many organs, including the heart [31], is implicated in DOXO-induced cardiac apoptosis [20] and is downregulated in DOXO-exposed human CPCs [14]. SIRT1 plays an important role in the development and progression of heart failure through the regulation of cell death/survival-related signalling [32, 33] and numerous evidence shows that molecules that activate SIRT1 are cardioprotective in several models of cardiovascular diseases, including DOXO-induced cardiotoxicity [24, 26, 34, 35]. Importantly, our recent study demonstrated that SIRT1 activation by resveratrol protects DOXO-exposed human CPCs and re-establishes their proper functions [24]. Results of the present work show that the prosurvival function of Bcl-2 and SIRT1, recovered by Ant34a, are critical in DOXO-treated rCPCs. Additionally, miR-34a induces cell senescence through the most commonly accepted p53-miR34a-SIRT1 axis with a positive feedback loop [36, 37]. Genotoxic stress activates p53, the strongest inducer of miR-34a that inhibits SIRT1 expression, which in turn inactivates p53 by its deacetylation. Cellular senescence is the main process that lead to functional failure of stem cells contributing to the onset and progression of heart failure [13, 14, 24, 38–40]. Data of the present investigation indicate that miR-34a pharmacological inhibition in DOXO exposed rCPCs significantly reduce both p53-acetylated forms according to SIRT1 increased levels. These molecular changes lead to an important attenuation of DOXO-induced cellular senescence as demonstrated by the reduction of p16^INK4a^ expression and SA-β-galactosidase activity. In conclusion, DOXO-induced apoptosis and senescence in rCPCs can be counteracted by miR-34a pharmacological repression. These results shed light on the complex pathways that underlie DOXO cardiac damage and propose miR-34a inhibition as a promising therapeutic approach for anthracycline-induced cardiotoxicity.

As on one hand the intrinsic ability of miR-34a to target different DOXO-related pathways makes its inhibition an attractive therapeutic perspective, on the other its clinical implications remain to be determined. The potential candidates for such cardioprotective strategy should be oncologic patients but antimiR treatment could interfere with antitumor activity of DOXO. Nevertheless, our results show that to reduce DOXO cytotoxic effect in tumor cells, an effective concentration of Ant34a needs to be 10-fold higher than the concentration that protects rCPCs. So theoretically, its potential to prevent or reduce anthracycline cardiotoxicity in oncologic patients should not influence antitumor therapy. In the view of discording studies that have reported a different role of miR-34a in modulating tumor progression [23, 41, 42], it could be suitable to address miR-34a inhibition exclusively to the heart. At the same time, miR-34a is the first miRNA entered into phase I clinical trials as emerging antitumor approach (NCT01829971 clinicaltrials.gov). However, taking into account our results, this therapy could theoretically increase the risk of cardiovascular side effects making necessary long-term studies.

Together with the need of an effective cardioprotective approach for DOXO-induced toxicity, the detection of earlier instrumental or biochemical markers of cardiac injury able to predict heart failure is of crucial importance. Recent evidences describe the dysregulation of miRNAs across the heart upon DOXO treatment [43–45] and importantly, miR-34a expression has been found upregulated in the heart of DOXO-cardiomyopathic mice [16]. In addition, miR-34a has been identified as a predictive circulating marker of future heart failure in patients after acute myocardial infarction [17]. Interestingly, the present work reveals that rat cardiac cells exposed to DOXO secrete increased levels of this miRNA. Therefore, the hypothesis that miR-34a could represent a possible circulating marker for DOXO-induced cardiotoxicity has been explored. Our results indicated that plasma of rats treated with DOXO is highly enriched in miR-34a and, although anthracycline administration upregulated miR-34a not only in the heart but also in liver, kidney and skeletal muscle, the cardiac contribution seems to be predominant. Moreover, it remains to verify if cells from other organs could release miR-34a after DOXO exposure thus concurring to secrete this miRNA in plasma. We documented remarkably higher miR-34a levels in plasma with respect to cardiac tissue of DOXO-cardiomyopathic rats. This finding could be explained by the extreme stability of circulating miRNAs that in our settings are packaged in exosomes.

To our knowledge, it is the first time that a miRNA is been recognized as a potential circulating biomarker associated to DOXO-induced cardiac damage. Only two studies had attempted to identify circulating miR-208 as a marker for DOXO-cardiotoxicity in breast cancer patients and in an animal model, but both failed to demonstrate their intention [46, 47]. In our settings, the animals were sacrificed one week after the last injection of DOXO (3 weeks). This time represents an early stage of the drug-induced cardiac damage, which progresses in the long term. In fact, the abnormalities in cardiac structure, myocyte performance and above all the regenerative properties of stem cells population found at 3 weeks after DOXO were markedly worsened at 6 weeks, impairing ventricular hemodynamics and affecting the animal survival as showed in the paper by De Angelis [13]. Circulating miR-34a increases in the animals treated with DOXO sacrificed at 3 weeks, but its levels could be also higher during, at the end or also upon DOXO administration. Therefore, the validity of miR-34a as DOXO-associated biomarker should be assessed with further studies to investigate how the time but also the dose of DOXO could influence this parameter. Moreover, clinical implications of miR-34a as biomarker of cardiac toxicity consequent to anthracycline therapy in oncologic patients should be carefully evaluated since its circulating levels could be dysregulated in some types of tumors [23] and its release could be also linked to tumor cell damage.

## MATERIALS AND METHODS

### Rat cardiac cells

rCPCs were isolated from 2-3 months old female Fisher 344 rat hearts (Harlan Laboratories), slightly modifying the protocol described by Smith AJ [48]. Briefly, hearts were rapidly dissected and washed in Ham's F12 medium supplemented with penicillin (100 U/ml), streptomycin (100 mg/ml) (Euroclone). Then the tissue was directly minced and dissociated in a sterile collagenase, type II solution (280 U/ml, Worthington) at 37°C for 40-60 minutes. Cell suspension was run through cell strainers with different pore diameters, respectively 100-μm and 40-μm, and subsequently subjected to serial centrifugations. Isolated cells were sorted for c-kit with rabbit anti-c-kit (Santa Cruz Biotechnology) and magnetic immunobeads (Miltenyi) as previously described [13, 48, 49]. c-kit positive cells were cultured and expanded in Ham's F12 medium, supplemented with 10% (v/v) fetal bovine serum (Euroclone), 5% (v/v) horse serum (Euroclone), 100 mM glutathione, 10U/ml erythropoietin (Sigma-Aldrich), 100 ug/ml bFGF (Peprotech). rCPCs were used for experiments from P3 to P6.

Cardiac fibroblasts were isolated from hearts finely minced and incubated in collagenase/dispase 1:1 buffer (1 mg/mL, 200 U/mL each) for several rounds of 20′ at 37°C until final samples dissociation. Low speed centrifugation was used to remove myocytes and debris. Cell suspension was obtained by several mashes separations (70 μm and 40 μm diameter pores) and centrifugation. Fibroblasts were plated and cultured in Ham's F12 medium, supplemented with 10% (v/v) fetal bovine serum, 5% (v/v) horse serum, 100 mM glutathione, 10 U/mL erythropoietin, 100 μg/mL bFGF. After twenty-four hours, floating cells were discarded and adherent cells, mainly fibroblasts, were cultured in the same medium.

Rat embryonic myoblasts (H9c2) were obtained from ATCC. Cells were cultured in DMEM medium (Euroclone, Italy), supplemented with 10% (v/v) fetal bovine serum.

Rat aortic endothelial cells (RAOECs) were obtained from ECACC and amplified in Rat Endothelial Cell Growth Medium (Cell Applications, Inc.)

Breast cancer cell line MCF7 were obtained from ATCC. Cells were cultured in DMEM medium, supplemented with 2 mM L-Glutamine; 10% (v/v) fetal bovine serum.

### Cell treatment

rCPCs, H9c2, fibroblasts and RAOECs were exposed to 0.5 μM DOXO (Adriblastina, Pfizer) for 24h. This concentration of DOXO is comparable with drug plasma levels in patients after standard infusion [20, 50].

To silence miR-34a, rCPCs were exposed for 24 hours to 10 nM has-miR-34a-5p mirVana miRna inhibitor (Ant34a) (Ambion). MCF7 were exposed for 24 hours to 5-10-30-60-100 nM Ant34a. MirVana miRNA inhibitor negative control (AntCTL) (Ambion) was used as control. Transfection of Ant34a or AntCTL was performed with Lipofectamine RNAiMAX Transfection Reagent (2 μl/ml) and Opti-MEM medium (Life Technologies) according to the manufacturer's instructions. The cells were then treated with 0.5 μM DOXO for 24h.

### Collection of CM

Cell culture media from treated rCPCs were centrifuged for 5′ at 200g and the supernatants were collected. H9c2, fibroblasts and RAOECs were exposed to rCPC CM for 48h.

### Cell viability assay

MTT (3-(4, 5-Dimethylthiazol-2-yl)-2, 5-diphenyltetrazolium bromide) assay was used to determine cell viability [51]. Yellow MTT is reduced to purple formazan when mitochondrial reductase enzymes are active. This conversion is directly related to the number of living cells. Optical density (OD) was measured at 540 nm with iMark microplate reader (Biorad).

### Cell proliferation

Cell proliferation was evaluated by Bromo-2′-deoxy-uridine (BrdU) Labeling and Detection Kit I (Roche). One hour before the end of the experiment, rCPCs were incubated with BrdU (10 μM). The cells were fixed and stained as suggested by manufacturer's instructions. Cell proliferation was determined by the percentage of BrdU-positive cells.

### TdT assay

Terminal deoxynucleotidyltransferase (TdT)–mediated dUTP nick end labeling assay was used for the detection of apoptosis by ApoAlert DNA fragmentation kit (Clontech). Cells were fixed with 4% paraformaldehyde and then stained as suggested by manufacturer's instructions. The number of apoptotic cells (TdT-positive) was counted blindly in randomly selected fields at 1000x magnification and expressed as a percentage of a total number of counted cells.

### Western blotting

Cells were resuspended in lysis buffer containing 50 mmol/L Tris-HCl (pH 7.4), 5 mmol/L EDTA, 250 mmol/L NaCl, 0.1% Triton X-100 and protease inhibitors (Sigma-Aldrich). 20 μg/lane of total cellular proteins were resolved by 10-12% SDS-polyacrylamide gel electrophoresis and transferred to a PVDF membrane (Thermo Fisher). Membranes were probed with primary antibodies against p16^INK4^, SIRT1, acetyl-p53^Lys370^, acetyl-p53^Lys381^ (Abcam), p53 (Cell Signaling), Bcl-2 (Sigma-Aldrich). Horseradish peroxidase-coupled secondary antibodies (Santa Cruz Biotechnology, Inc.) were used to detect primary antibody and developed by a chemiluminescence-based detection system (ECL, Amersham). Loading conditions were determined with α-Tubulin (Sigma-Aldrich). Images were obtained by ChemiDoc-it 500 Imaging System and analyzed by Vision Works LS analysis software (UVP).

### Senescence-associated β-galactosidase

Senescent cells were detected by senescence-associated β-galactosidase (SA-β-gal) activity using the artificial substrate 5-bromo-4-chloro-3-indolyl β-D-galactoside (X-gal) (Promega) [52]. Briefly, cells were fixed for 5 min in 2% formaldehyde/0.2% glutaraldehyde (Sigma-Aldrich) and incubated at 37°C with fresh SA-β-Gal staining solution containing 1 mg/ml X-gal. Cell senescence was determined by the percentage of β-gal positive cells.

### Quantitative real-time PCR

Total RNA was extracted from rCPCs by Trizol according to the manufacturer's instructions. Both cDNA synthesis and PCR were performed simultaneously by using the SuperScript III Platinum SYBR Green One-Step qRT-PCR Kit (Invitrogen). Quantitative real-time PCR (qPCR) was carried out using the CFX96 Real-time system (Bio-Rad). The transcript levels of SIRT1 and Bcl-2 were detected and the housekeeping gene encoding hypoxanthine phosphoribosyltransferase (HPRT) was used as internal control for mRNA expression studies. Relative expression was calculated using the comparative cycle threshold (Ct) method (2^−ΔΔCt^).

### Quantitative real-time PCR of miR-34a from cells and tissues

MiRNAs were extracted from cells by MirVana miRNA Isolation Kit (Ambion) according to the manufacturer's instructions. MiRNAs were also isolated from organs by miRCury RNA Isolation Kit (Exiqon) according to the manufacturer's instructions. Reverse-transcription (RT) were performed by TaqMan MicroRNA Reverse Transcription kit (Applied Biosystem) and GeneAmp PCR System 9700. qPCR were performed by using the TaqMan Universal PCR Master Mix (Applied Biosystem) using the CFX96 Real-time system (Bio-Rad). RT and qPCR used TaqMan® MicroRNA Assays (Applied Biosystem). Levels of miR-34a were detected and were normalized byRNU6B (U6) as internal control for miRNAs expression studies. Relative expression was calculated using the comparative cycle threshold (Ct) method (2^−^^ΔΔ^^Ct^).

### Quantitative real-time PCR of miR-34a from cell culture media, rat plasma and exosomes

Cell culture media were collected after cell treatment and concentrated by Centrifugal Filter Units (Millipore). Rat blood samples were collected and centrifuged to obtain plasma. MiRNAs were extracted from cell culture media and from rat plasma by miRNeasy Serum/Plasma Kit (Qiagen) and from circulating exosomes by exoRNeasy Serum/Plasma Midi Kit(Qiagen) according to the manufacturer's instructions. RT were performed by miScript II RT kit (Qiagen) and GeneAmp PCR System 9700. qPCR were performed by using the miScript SybrGreen PCR Kit using the CFX96 Real-time system (Bio-Rad). qPCR used miScript Primer Assay. MiR-34a levels were detected and normalized by using *C. elegans* miR-39 miRNeasy Serum/Plasma Spike-in control. Relative expression was calculated using the comparative cycle threshold (Ct) method (2^−^^ΔΔ^^Ct^).

### Cloning and luciferase reporter assay for pGL3-SIRT1-3′UTR vector

pGL3-SIRT1-3′UTR vectors (Supplementary Table 1) were obtained as previous described [53] and transfected into MCF7 cells using using Lipofectamine 2000 Transfection Reagent (Invitrogen), following supplier's instructions. 1 μg pGL3-Sirt1-3′UTR plasmids plus mimic-miR-34a-5p (100 nM and 200 nM) (pre-miR-34a) or mimic-miR-CTL (200 nM) (pre-miR-CTL) were used. For normalization, 1 μg pMAX-GFP vector was transfected into all the samples. Luciferase activity was measured after 24 and 48 h from pGL3-SIRT1-3′UTR transfections as reported protocol [53]. The results were expressed as normalized luciferase activity.

### Fluorescent in situ hybridization (FISH)

MiR-34a was detected by FISH according to protocol previously described [54] and modified as follow. Tissues sections were digested by 0.5μg/ml proteinase-K for 10′ at RT. miRCURY LNA detection probe hsa-miR-34a, 5′-DIG and 3′-DIG labeled and control probe LNA U6 snRNA, 5′-DIG labeled (Exiqon) were used at 50 nM and 10 nM respectively and denatured as suggested by manufacturer's instructions. To detect signal Anti-Digoxigenin antibody (Abcam) and TSA Fluorescein Plus evaluation kit (Perkin Elmer) were used. rCPCs were identified by c-kit (Santa Cruz Biotechnology), myocytes were labeled with α-sarcomeric actin (Sigma-Aldrich) and nuclei were stained with DAPI (Sigma-Aldrich). Samples were analyzed with a Zeiss LSM700 confocal microscope.

### *In vivo* studies

The present study conforms to the National Ethical Guidelines (Italian Ministry of Health; D.L.vo 26, March 4, 2014) and has been performed upon approval of local ethics committee. Cardiomyopathy was induced in 2-3 months old female Fisher 344 rats (Harlan Laboratories) (n=10) by 6 intraperitoneal injections of 2.5 mg/kg of DOXO over a period of 2 weeks to reach a cumulative dose of 15 mg/kg [13, 17, 25]. Control rats (n=5) were injected with saline solution. At 3 weeks after the first injection of DOXO, animals were anesthetized with ketamine (100 mg/kg b.w., i.p.) and echocardiographic parameters were analysed with a high resolution Micro-Ultrasound system System (Vevo 770, VisualSonics Inc.) equipped with a 25- MHz linear transducer [55, 56]. FS and EF were calculated. The animals were sacrificed and organs and blood samples were collected.

### Data analysis and statistics

Results are presented as mean ± SD. All data were analyzed with a GraphPad Prism version 5.01 statistical software package (GraphPad). Significance was determined by Student's t test and by ANOVA with Bonferroni post-test. All P values are two-sided and P<0.05 was considered to be significant.

## SUPPLEMENTARY FIGURES AND TABLE


