# Supplementary material for: MicroRNA-34a regulates doxorubicin-induced cardiotoxicity in rat
Source: Oncotarget. 2016 Aug 22;7(38):62312–26. doi: 10.18632/oncotarget.11468 (PMC5308729; doi:10.18632/oncotarget.11468)
Supplement: Supplementary file 1 [file oncotarget-07-62312-s001.pdf]

MicroRNA-34a regulates doxorubicin-induced cardiotoxicity in rat

SUPPLEMENTARY FIGURES AND TABLE

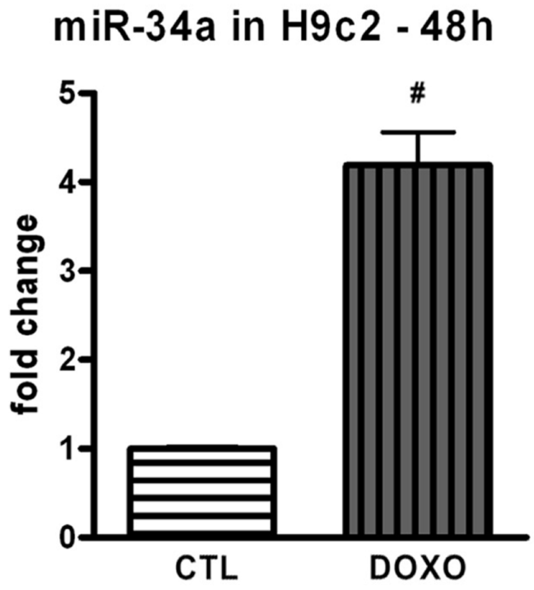

Supplementary Figure S1: miR-34a in H9c2 exposed to DOXO for 48h. qPCR analysis of miR-34a levels in H9c2 exposed to DOXO for 48h. Results are indicated as mean ± SD. MiRNA expression is reported as fold change vs CTL. *p<0.05 vs CTL.

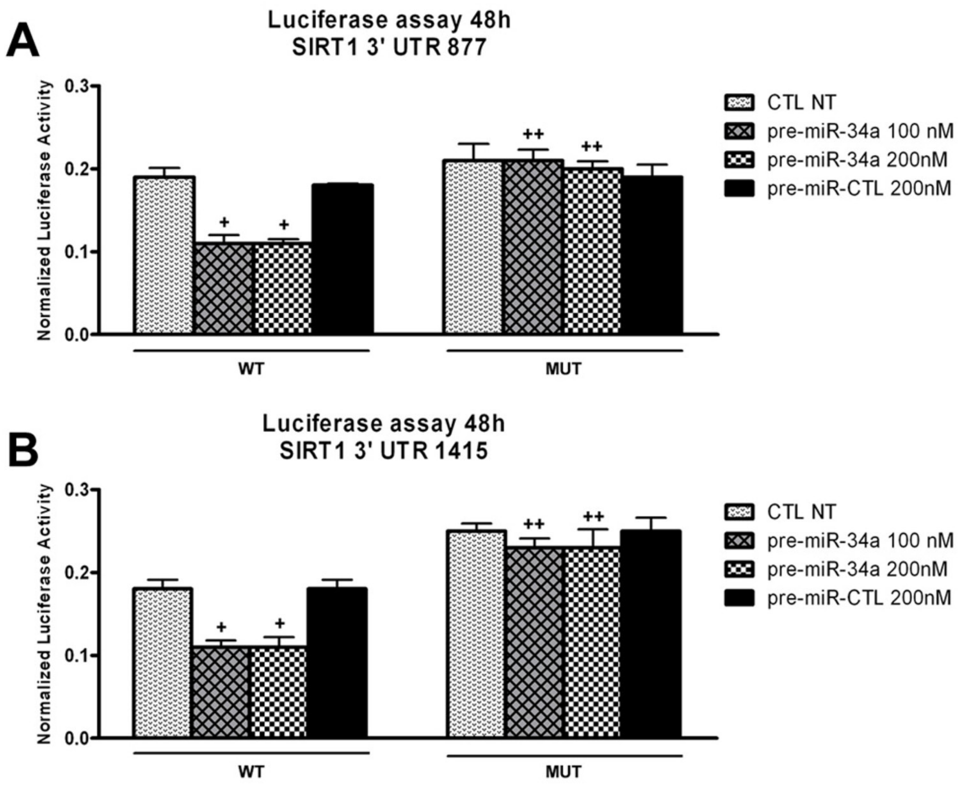

Supplementary Figure S2: Luciferase activity assay at 48h. A. Luciferase activity assay at 48h for SIRT1 3'UTR 877 and **B.** SIRT1 3'UTR 1415. Results are expressed as normalized luciferase activity and are indicated as mean ± SD. $^{+}p<0.05$ vs CTL NT; $^{++}p<0.05$ vs corresponding WT conditions. CTL NT: no treated control; WT: Wild Type; MUT: mutated

Supplementary Table S1: 3'UTR sequences of *SIRT1* gene

3'UTR sequences of *SIRT1* gene	Sequences
**WT 3'UTR 1415 FW**	cta gag ctg agc aca ccc agc tag gac cat tac tgc caa cac cca gct agg acc att act gcc aac acc cag cta gga cca tta ctg cca t
**WT 3'UTR 1415 REV**	cta gat ggc agt aat ggt cct agc tgg gtg ttg gca gta atg gtc cta gct ggg tgt tgg cag taa tgg tcc tag ctg ggt gtg ctc agc t
**MUT 3'UTR 1415 FW**	cta gag ctg agc aca cca aat tag gac cat tac tgc caa cac caa att agg acc att act gcc aac acc aaa tta gga cca tta ctg cca t
**MUT 3'UTR 1415 REV**	cta gat ggc agt aat ggt cct aat ttg gtg ttg gca gta atg gtc cta att tgg tgt tgg cag taa tgg tcc taa ttt ggt gtg ctc agc t
**WT 3'UTR 877 FW**	cta gag ctg agc tcc aca agt att aaa ctg cca tcc aca agt att aaa ctg cca tcc aca agt att aaa ctg cca t
**WT 3'UTR 877 REV**	cta gat ggc agt tta ata ctt gtg gat ggc agt tta ata ctt gtg gat ggc agt tta ata ctt gtg gag ctc agc t
**MUT 3'UTR 877 FW**	cta gag ctg agc tcc aaa cat att aaa ctg cca tcc aaa cat att aaa ctg cca tcc aaa cat att aaa ctg cca t
**MUT 3'UTR 877 REV**	cta gat ggc agt tta ata tgt ttg gtt ggc agt tta ata tgt ttg gtt ggc agt tta ata tgt ttg gtg ctc agc t

Sequences of *SIRT1* 3'UTR cloned into pGL3 control vector inserted in triple copy including XbaI restriction sites ends for cloning in pGL3-control vector (t cta ga) and unique Bpl1 restriction sites for positive control of insertion (gct cag c).WT: Wild Type; MUT: mutated; FW: forward; REV: reverse.
